# Hypertensive Emergency Team-Based Learning

**DOI:** 10.21980/J8BP90

**Published:** 2024-04-30

**Authors:** Khoa Nguyen, Jordan Gawon Shin, Jessica Andrusaitis

**Affiliations:** * University of California, Irvine, Department of Emergency Medicine, Orange, CA; ^ University of California, Irvine, School of Medicine, Irvine, CA

## Abstract

**Audience:**

The target audiences for this team-based learning (TBL) activity are resident physicians and medical students.

**Introduction:**

According to the Centers for Disease Control and Prevention (CDC), nearly half of the adults in the United States have hypertension,^1^ which is a leading cause of cardiovascular disease and premature death.^2^ In extreme cases, patients may present in hypertensive emergencies, defined as an acute, marked elevation of systolic blood pressure >180mmHg or diastolic blood pressure >120mmHg with evidence of organ dysfunction.^3^,^4^ Patients presenting to the emergency department (ED) with symptoms of hypertensive emergencies must be promptly diagnosed and treated to prevent further morbidity and mortality. This TBL utilizes four clinical cases to educate resident physicians and medical students not only on the recognition of hypertensive emergencies, but also on the workup, management, and disposition of patients who present to the ED with hypertension.

**Educational Objectives:**

By the end of this TBL session, learners should be able to: 1) define features of asymptomatic hypertension versus hypertensive emergency, 2) discuss which patients with elevated blood pressure may require further diagnostic workup and intervention, 3) identify a differential diagnosis for patients presenting with elevated blood pressures, 4) recognize the features of different types of end-organ damage, 5) review an algorithm for the pharmacologic management of hypertensive emergencies, 6) indicate dosing and routes of various anti-hypertensive medications, 7) choose the appropriate treatment for a patient who is hypertensive and presenting with flash pulmonary edema, 8) identify an aortic dissection on computed tomography (CT), 9) choose the appropriate treatment for a patient who is hypertensive and presenting with an aortic dissection, 10) identify intracranial hemorrhage on CT, 11) choose the appropriate treatment for a patient who is hypertensive and presenting with an intracranial hemorrhage, and 12) describe the intervention for warfarin reversal.

**Educational Methods:**

This is a classic TBL that includes an individual readiness assessment test (iRAT), a multiple-choice group readiness assessment test (gRAT), and a group application exercise (GAE).

**Research Methods:**

Learners and instructors were given the opportunity to provide verbal feedback after completion of the TBL. Learners included senior medical students and first-, second-, and third-year emergency-medicine residents. Learners were specifically asked if they felt the cases were educational, relevant, and useful to their training.

**Results:**

Six resident physicians and three medical students volunteered their verbal feedback, and agreed when they were specifically asked if the cases were educational, relevant, and useful to their training. The same learners also agreed when asked if they felt the TBL was a more enjoyable activity than a direct lecture to refresh their knowledge and skills. One instructor observed that interns and medical students were generally able to reach a correct diagnosis; however, they seemed to struggle more with describing appropriate pharmacologic interventions when compared to more senior learners.

**Discussion:**

Hypertension is a common complaint and incidental finding in patients presenting to the ED. Given its non-specific value, it can be a difficult topic for the novice healthcare provider to master. The differential diagnosis for a patient presenting with hypertension is vast, ranging from benign to emergent, and can sometimes necessitate minimal to substantial workups. Thus, this TBL is a useful, relevant, and effective exercise for residents-in-training to review and understand the management of hypertension.

**Topics:**

Hypertension, hypertensive emergency, asymptomatic hypertension, flash pulmonary edema, aortic dissection, intracranial hemorrhage, warfarin reversal, team-based learning.

## USER GUIDE

[Table t1-jetem-9-2-t1]

**Table t1-jetem-9-2-t1:** 

List of Resources:	
Abstract	1
User Guide	3
Learner Materials	6
iRAT	6
gRAT	8
GAE	10
Instructor Materials	20
RAT Key	21
GAE Key	23

**Learner Audience:**

Medical Students, Interns, Junior Residents, Senior Residents

**Time Required for Implementation:**

Instructor Preparation: 60–90 minutes.

Learner Responsible Content: 0–60 minutes.

In-Class Time: 60–90 minutes.

**Recommended Number of Learners per Instructor:**

1–20 learners

**Topics:**

Hypertension, hypertensive emergency, asymptomatic hypertension, flash pulmonary edema, aortic dissection, intracranial hemorrhage, warfarin reversal, team-based learning.

**Objectives:**

By the end of this team-based learning (TBL), learners should be able to:

1. Define features of asymptomatic hypertension versus hypertensive emergency.
2. Discuss which patients with elevated blood pressure may require further diagnostic workup and intervention.
3. Identify a differential diagnosis for patients presenting with elevated blood pressures.
4. Recognize the features of different types of end-organ damage.
5. Review an algorithm for the pharmacologic management of hypertensive emergencies.
6. Indicate medication dosing and routes of various anti-hypertensive medications.
7. Choose the appropriate treatment for a patient who is hypertensive and presenting with flash pulmonary edema.
8. Identify an aortic dissection on computed tomography (CT).
9. Choose the appropriate treatment for a patient who is hypertensive and presenting with an aortic dissection.
10. Identify intracranial hemorrhage on CT.
11. Choose the appropriate treatment for a patient who is hypertensive and presenting with an intracranial hemorrhage.
12. Describe the intervention for warfarin reversal.

### Linked objectives, methods and results

Hypertension is a common complaint and incidental finding in patients presenting to the emergency department (ED). Given its non-specific value, it can be a difficult topic for the novice emergency medicine resident to master. The differential diagnosis for a patient presenting with hypertension is vast, ranging from benign to emergent, and can sometimes necessitate minimal to substantial workups. Thus, this TBL is a useful, relevant, and effective exercise for residents in training to review and understand the management of hypertension by working through cases, including asymptomatic hypertension versus hypertensive emergency (objectives 1, 2, 3, and 4), a patient with flash pulmonary edema (objectives 5, 6, and 7), a patient presenting with an aortic dissection (objectives 6, 7, 8, and 9), and a patient on anticoagulation presenting with an intracranial hemorrhage (objectives 6, 7, 10, 11, and 12).

### Learner Responsible Content (LRC)

- American College of Emergency Physicians. Asymptomatic hypertension: A scientific statement from the American College of EmergencyPhysicians*. Ann Emerg Med*. 2013;62(1), 1–18. doi:10.1016/j.annemergmed.2013.01.017
- Barlow A, Barlow B. Hypertensive emergency: pearls and pitfalls for the ed physician. emDOCs.net. 2020. At: http://www.emdocs.net/hypertensive-crisis-pearls-and-pitfalls-for-the-ed-physician/
- Pickens A. Em in 5: hypertensive emergency treatment. emDOCs.net. 2018. https://www.emdocs.net/em-in-5-hypertensive-emergency-treatment/

### Recommended pre-reading for instructor

- American College of Emergency Physicians. Asymptomatic hypertension: A scientific statement from the American College of Emergency Physicians. *Ann Emerg Med*. 2013; 62(1):1–18. doi:10.1016/j.annemergmed.2013.01.017
- Gheorghiade M, Filippatos G, De Luca L, Burnett J. Congestion in acute heart failure syndromes: an essential target of evaluation and treatment. *Am J Med*. 2006;*119*(12). https://doi.org/10.1016/j.amjmed.2006.09.011
- Greenberg SM, Ziai WC, Cordonnier C, et al. 2022 guideline for the management of patients with spontaneous intracerebral hemorrhage: A guideline from the AHA/ASA. *Stroke.* 2022; 53(7), e282–e361. At: doi:10.1161/STR.0000000000000407
- Hiratzka LF, Bakris GL, Beckman JA, et al. Guidelines for the diagnosis and management of patients with thoracic aortic disease: a report of the American College of Cardiology Foundation/American Heart Association Task Force on Practice Guidelines, American Association for Thoracic Surgery, American College of Radiology, American Stroke Association, Society of Cardiovascular Anesthesiologists, Society for Cardiovascular Angiography and Interventions, Society of Interventional Radiology, Society of Thoracic Surgeons, and Society for Vascular Medicine. *Circ*. 2010;121:e266–e369.

### Results and tips for successful implementation

This TBL was administered during a resident didactics session at University of California, Irvine, Department of Emergency Medicine. Although originally written to be implemented in a paper format, a digitized version of this activity was created on Microsoft Forms and utilized for this particular session. One emergency-medicine/medical-education fellow acted as the instructor. Learners included medical students and first-, second-, and third-year emergency-medicine residents. Learners and instructors were given the opportunity to provide verbal feedback after completion of the TBL. Learners were specifically asked if they felt the cases were educational, relevant, and useful to their training.

Six resident physicians and three medical students volunteered their verbal feedback and agreed when they were specifically asked if they felt the cases were educational, relevant, and useful to their training. The same learners also agreed when asked if they felt the TBL was a more enjoyable activity than a direct lecture to refresh their knowledge and skills. Some senior learners found the cases to be straight-forward. The instructor observed that interns and medical students seemed to struggle more with describing appropriate pharmacologic interventions when compared to junior and senior learners.

Moreover, the authors noted that creating a digitized version of this TBL allowed for greater opportunities for its implementation, such as in remote didactics or in asynchronous curriculums. We believe that this would greatly benefit neurodivergent learners and those who are unable to attend didactics sessions. This online version where learners can receive immediate feedback on their answers could also be utilized as a resource for independent studying.

#### Instructions for Implementation

1. (OPTIONAL) Prior to the session, ideally twenty-four hours in advance, email the Learner Responsible Content to the learners.
2. Introduce session.
3. Administer one individual readiness assessment test (iRAT) per learner and have them complete it individually.
4. Administer one group readiness assessment test (gRAT) per group of 2–5 learners and have them complete it in individual groups.
5. Instructor reviews answers for gRAT.
6. Administer the group application exercise (GAE) to each group.
7. Instructor reviews answers for GAE. We recommend calling on individual groups to answer these questions. Instructor manual will have more in-depth answers in case of questions posed by learners.

### Content

- iRAT
  ○ https://forms.office.com/Pages/ShareFormPage.aspx?id=dGqfIFMJpE2HKkcxkbtXXbR94NwoakBJkvy9eJcaW6xUOFA3ODVJQzVBT0dFNkxRUFg0N09ITkhQMy4u&sharetoken=nMyTLa96ALEko8g9DyEk
- gRAT
  ○ https://forms.office.com/Pages/ShareFormPage.aspx?id=dGqfIFMJpE2HKkcxkbtXXbR94NwoakBJkvy9eJcaW6xUMkkyQlVMN0o1UTNJSTlWVDkzM1VEMFgyMi4u&sharetoken=kad4X9MFrwoGqDXlBe4E
- GAE
  ○ https://forms.office.com/Pages/ShareFormPage.aspx?id=dGqfIFMJpE2HKkcxkbtXXbR94NwoakBJkvy9eJcaW6xUMU1ZR0xJNUszVkpGOTBCRFJMUjlIN1ZZUy4u&sharetoken=7wpTMKE2yTZSHzjERAoR
- RAT Key
- GAE Key

## Figures and Tables

**Figure f1-jetem-9-2-t1:**
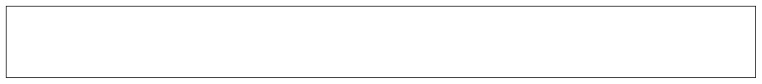


**Figure f2-jetem-9-2-t1:**
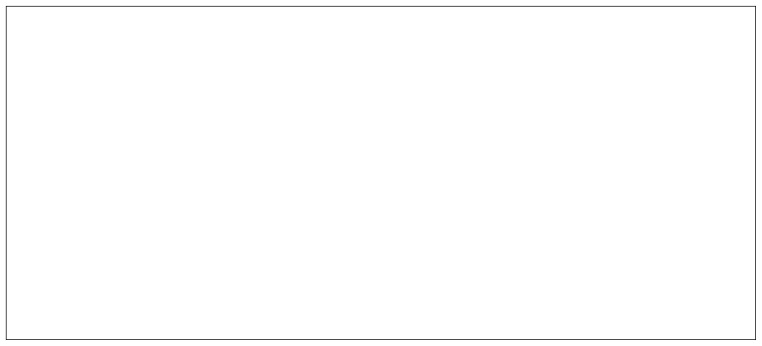


**Table t2-jetem-9-2-t1:** 

Complete Blood Count (CBC):	Basic Metabolic Panel (BMP):
White blood cells (WBC) 10,000/mm^3^	Sodium (Na) 135 mEq/L
Hemoglobin (Hgb) 12.0g/dL	Potassium (K) 5.6 mEq/L
Hematocrit (Hct) 41%	Chloride (Cl) 100 mEq/L
Platelets (Plt) 250 ×10^3^/mm^3^	Bicarbonate (HCO_3_) 24 mEq/L
	Blood urea nitrogen (BUN) 15 mEq/L
	Creatinine (Cr) 1.0 mEq/L
	Glucose (Glu) 132 mEq/L

**Figure f3-jetem-9-2-t1:**
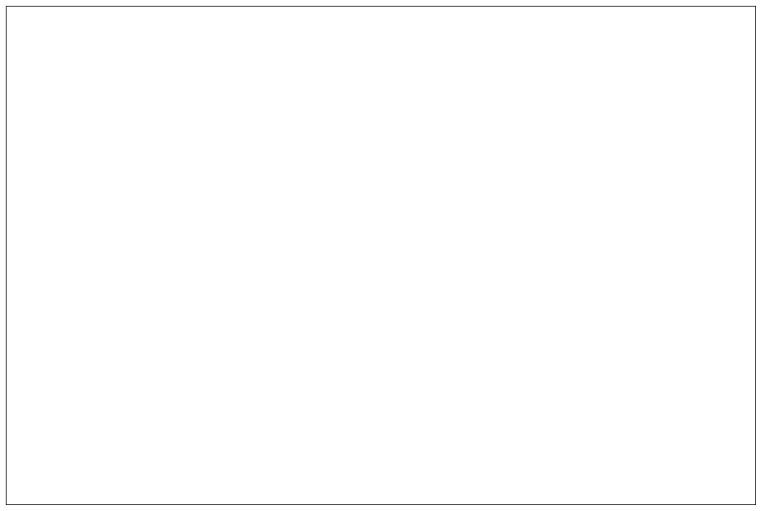


**Figure f4-jetem-9-2-t1:**
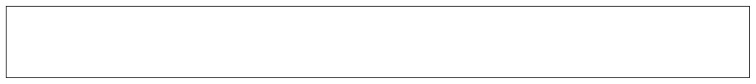


**Figure f5-jetem-9-2-t1:**
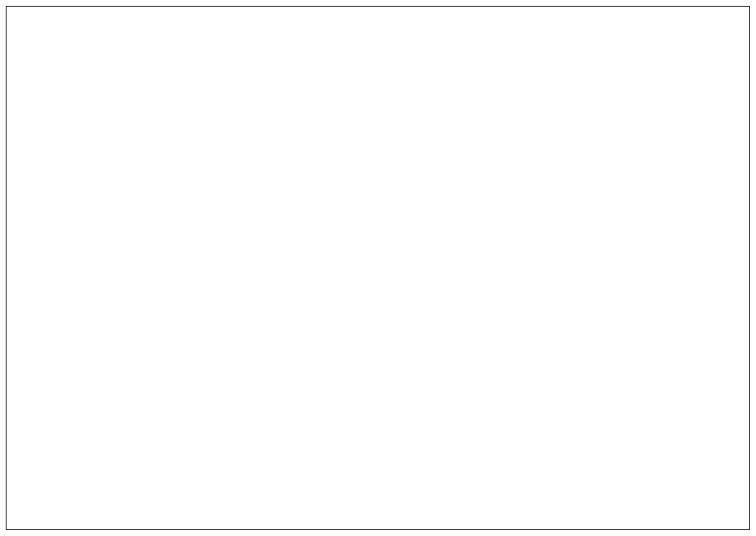


**Figure f6-jetem-9-2-t1:**
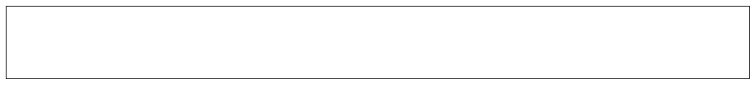


**Figure f7-jetem-9-2-t1:**
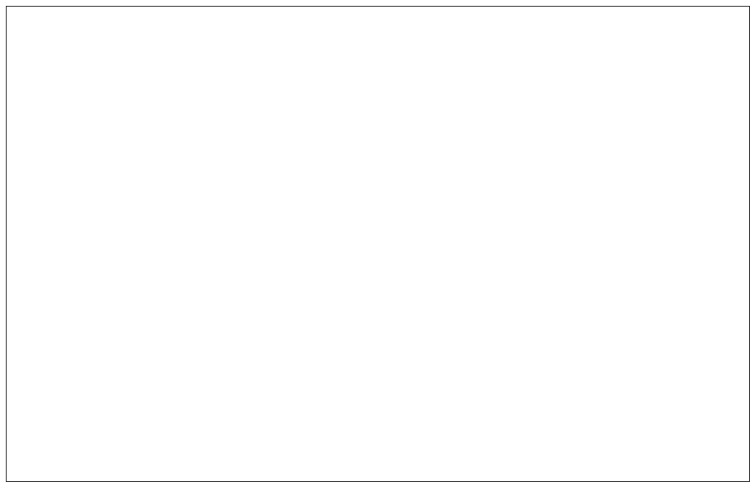


**Table t3-jetem-9-2-t1:** 

CBC:	BMP:
WBC 8,000/mm^3^	Na 139 mEq/L
Hgb 14 g/dL	K 3.7 mEq/L
Hct 45%	Cl 101 mEq/L
Plt 265 ×10^3^/mm^3^	HCO_3_ 23 mEq/L
	BUN 30 mEq/L
	Cr 2.0 mEq/L
	Glu 132 mEq/L

**Figure f8-jetem-9-2-t1:**
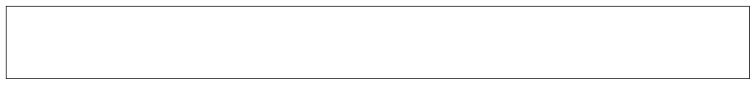


**Figure f9-jetem-9-2-t1:**
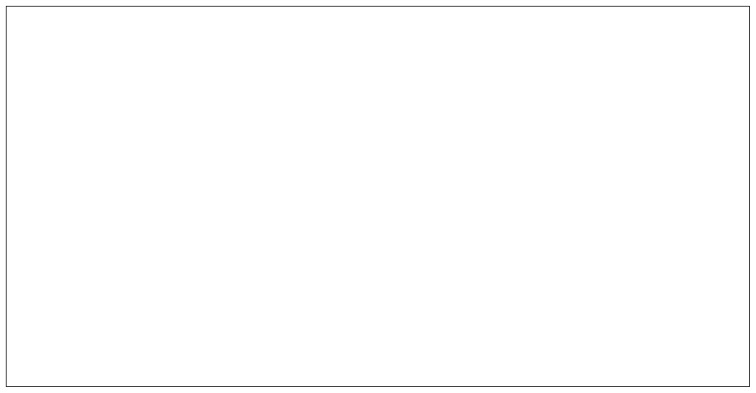


**Figure:**
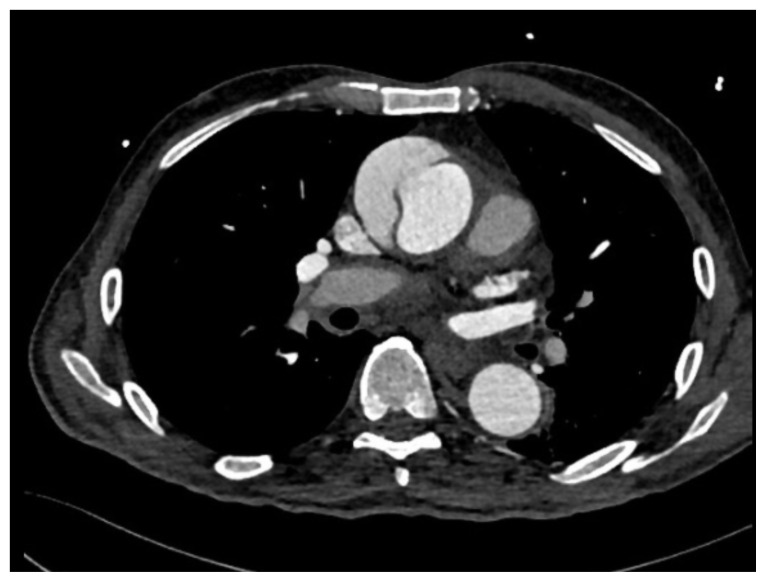


**Figure f11-jetem-9-2-t1:**
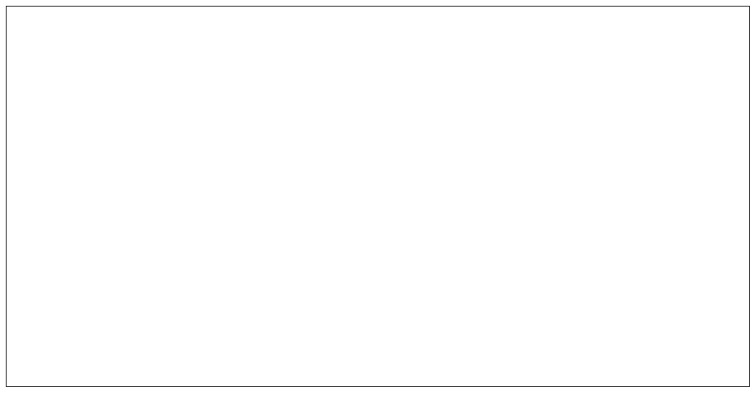


**Figure f12-jetem-9-2-t1:**
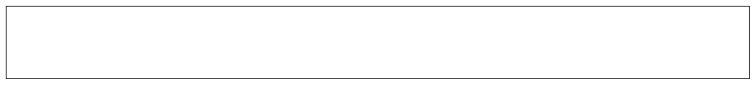


**Figure f13-jetem-9-2-t1:**
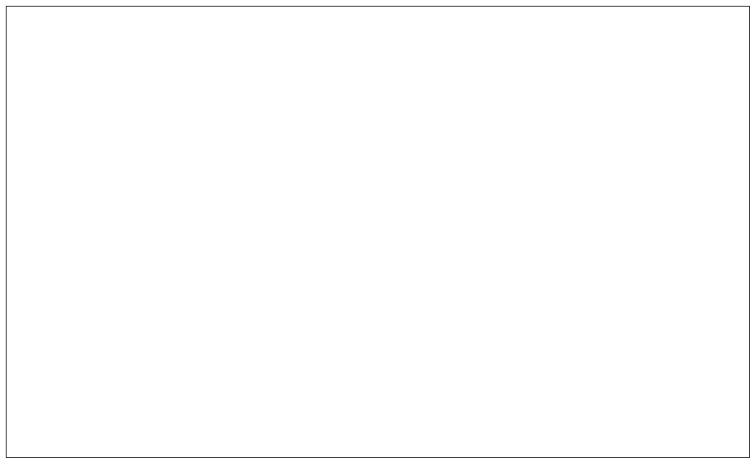


**Table t4-jetem-9-2-t1:** 

CBC:	BMP:
WBC 10,000/mm^3^	Na 141 mEq/L
Hgb 13 g/dL	K 3.8 mEq/L
Hct 47%	Cl 102 mEq/L
Plt 220 ×10^3^/mm^3^	HCO_3_ 22 mEq/L
	BUN 15 mEq/L
	Cr 1.2 mEq/L

**Figure f14-jetem-9-2-t1:**
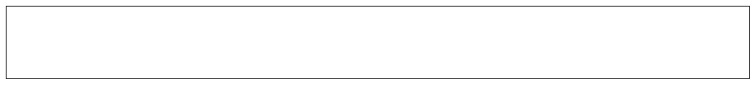


**Figure f15-jetem-9-2-t1:**
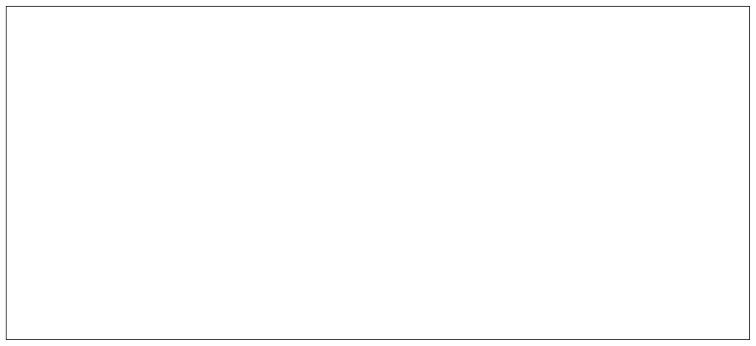


**Figure:**
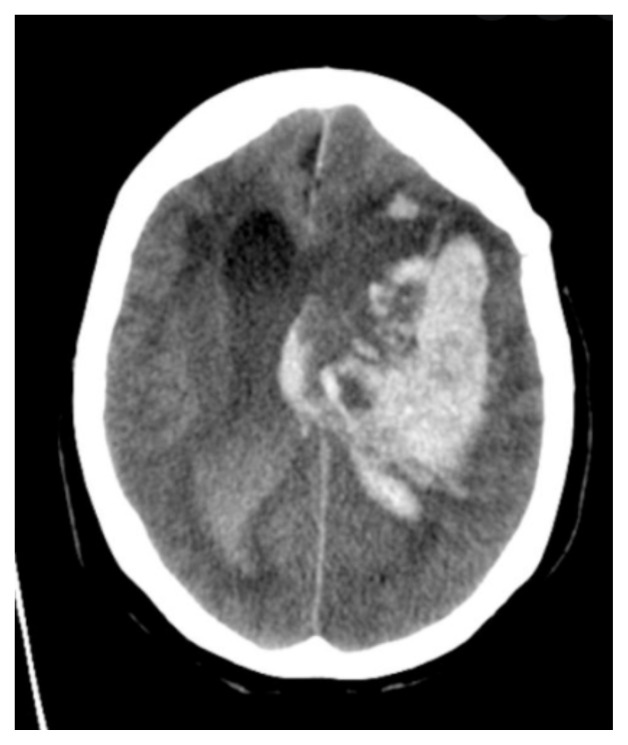


**Figure f17-jetem-9-2-t1:**
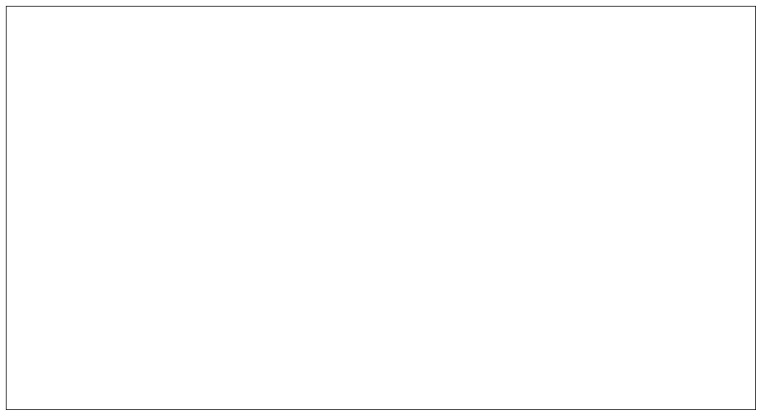


**Figure f18-jetem-9-2-t1:**
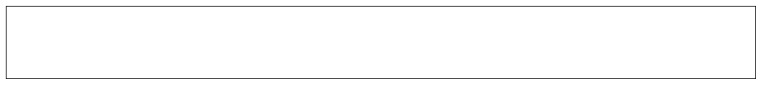


**Figure f19-jetem-9-2-t1:**
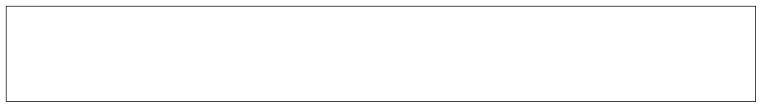


**Figure f20-jetem-9-2-t1:**
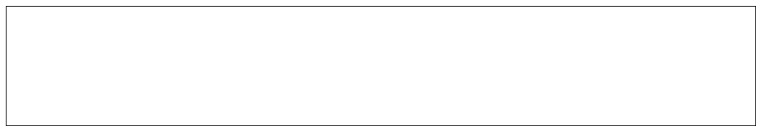


**Figure f21-jetem-9-2-t1:**
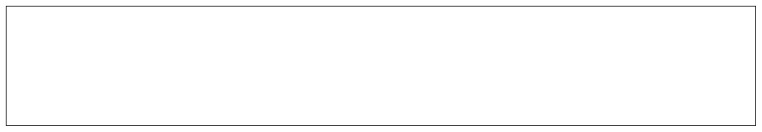


**Table t5-jetem-9-2-t1:** 

Complete Blood Count (CBC):	Basic Metabolic Panel (BMP):
White blood cells (WBC) 10,000/mm^3^	Sodium (Na) 135 mEq/L
Hemoglobin (Hgb) 12.0g/dL	Potassium (K) 5.6 mEq/L
Hematocrit (Hct) 41%	Chloride (Cl) 100 mEq/L
Platelets (Plt) 250 ×10^3^/mm^3^	Bicarbonate (HCO_3_) 24 mEq/L
	Blood urea nitrogen (BUN) 15 mEq/L
	Creatinine (Cr) 1.0 mEq/L
	Glucose (Glu) 132 mEq/L

**Table t6-jetem-9-2-t1:** 

CBC:	BMP:
WBC 8,000/mm^3^	Na 139 mEq/L
Hgb 14 g/dL	K 3.7 mEq/L
Hct 45%	Cl 101 mEq/L
Plt 265 ×10^3^/mm^3^	HCO_3_ 23 mEq/L
	BUN 30 mEq/L
	Cr 2.0 mEq/L
	Glu 132 mEq/L

**Figure:**
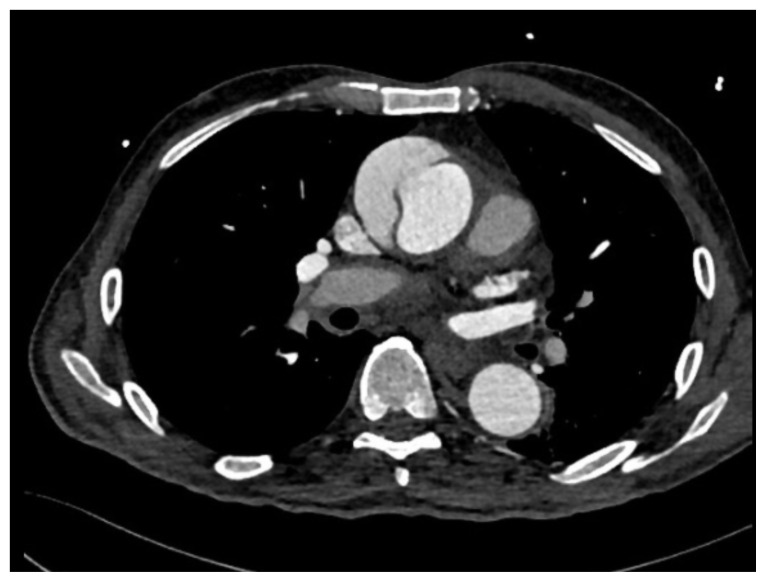


**Table t7-jetem-9-2-t1:** 

CBC:	BMP:
WBC 10,000/mm^3^	Na 141 mEq/L
Hgb 13 g/dL	K 3.8 mEq/L
Hct 47%	Cl 102 mEq/L
Plt 220 ×10^3^/mm^3^	Bicarb 22 mEq/L
	BUN 15 mEq/L
	Cr 1.2 mEq/L
	Glu 121 mEq/L

**Figure:**
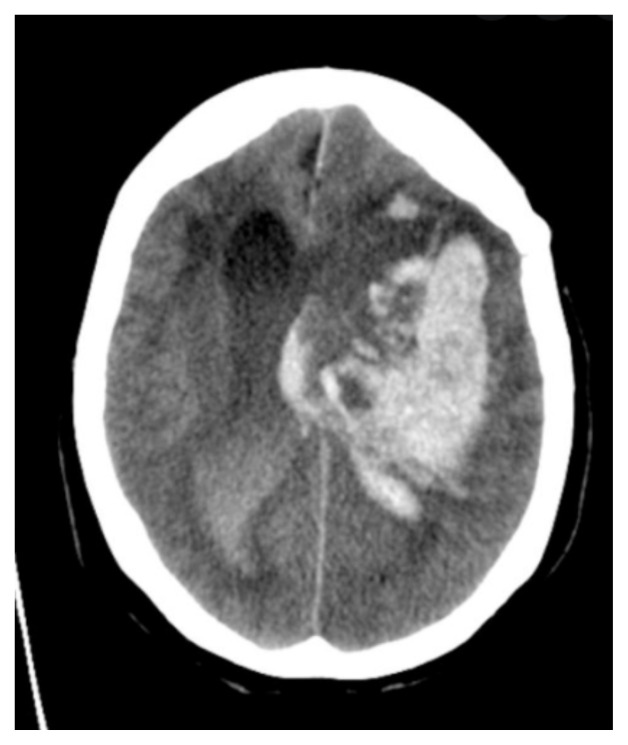


## LEARNER MATERIALS

### Hypertensive Emergency TBL: individual Readiness Assessment Test (iRAT)

**Digital:** https://forms.office.com/Pages/ShareFormPage.aspx?id=dGqfIFMJpE2HKkcxkbtXXbR94NwoakBJkvy9eJcaW6xUOFA3ODVJQzVBT0dFNkxRUFg0N09ITkhQMy4u&sharetoken=nMyTLa96ALEko8g9DyEk

1. The 2013 ACEP Clinical Policy regarding asymptomatic elevated blood pressure defines markedly elevated blood pressure as:
  - Systolic blood pressure greater than or equal to 160 mmHg or diastolic blood pressure greater than or equal to 100 mmHg
  - Systolic blood pressure greater than or equal to 180 mmHg or diastolic blood pressure greater than or equal to 110 mmHg
  - Systolic blood pressure greater than or equal to 220 mmHg or diastolic blood pressure greater than or equal to 180 mmHg
2. The 2013 ACEP Clinical Policy regarding asymptomatic elevated blood pressure states that routine screening for acute target organ injury (eg, serum creatinine, urinalysis, ECG) is not required.
  - True
  - False
3. According to the 2013 ACEP Clinical Policy regarding asymptomatic elevated blood pressure, routine ED medical intervention for markedly elevated blood pressure is:
  - Supported
  - Not required
  - Considered to have potential harm
4. According to the 2013 ACEP Clinical Policy regarding asymptomatic elevated blood pressure, rapid lowering of elevated blood pressure in the asymptomatic patient is:
  - Supported
  - Not required
  - Considered to have potential harm
5. The 2013 ACEP Clinical Policy recommends consideration of treating asymptomatic hypertension in all of the following populations except:
  - Patients with poor follow up
  - Patients with limited access to care
  - Patients less than 18 years old
  - Black patients
  - Elderly patients
6. According to the 2013 ACEP Clinical Policy regarding asymptomatic elevated blood pressure, which screening test would most likely affect disposition?
  - ECG
  - Creatinine
  - Troponin
  - None of these alters disposition
7. Which of the following management steps does the 2013 ACEP Clinical Policy recommend for asymptomatic markedly elevated blood pressure?
  - Screening for kidney disease
  - Referring for outpatient follow-up
  - Observation and repeat blood pressure measurement
  - Rapid lowering of mean arterial pressure at 25%

### Hypertensive Emergency TBL: group Readiness Assessment Test (gRAT)

**Digital:** https://forms.office.com/Pages/ShareFormPage.aspx?id=dGqfIFMJpE2HKkcxkbtXXbR94NwoakBJkvy9eJcaW6xUMkkyQlVMN0o1UTNJSTlWVDkzM1VEMFgyMi4u&sharetoken=kad4X9MFrwoGqDXlBe4E

1. The 2013 ACEP Clinical Policy regarding asymptomatic elevated blood pressure defines markedly elevated blood pressure as:
  - Systolic blood pressure greater than or equal to 160 mmHg or diastolic blood pressure greater than or equal to 100 mmHg
  - Systolic blood pressure greater than or equal to 180 mmHg or diastolic blood pressure greater than or equal to 110 mmHg
  - Systolic blood pressure greater than or equal to 220 mmHg or diastolic blood pressure greater than or equal to 180 mmHg
2. The 2013 ACEP Clinical Policy regarding asymptomatic elevated blood pressure states that routine screening for acute target organ injury (eg, serum creatinine, urinalysis, ECG) is not required.
  - True
  - False
3. According to the 2013 ACEP Clinical Policy regarding asymptomatic elevated blood pressure, routine ED medical intervention for markedly elevated blood pressure is:
  - Supported
  - Not required
  - Considered to have potential harm
4. According to the 2013 ACEP Clinical Policy regarding asymptomatic elevated blood pressure, rapid lowering of elevated blood pressure in the asymptomatic patient is:
  - Supported
  - Not required
  - Considered to have potential harm
5. The 2013 ACEP Clinical Policy recommends consideration of treating asymptomatic hypertension in all of the following populations except:
  - Patients with poor follow up
  - Patients with limited access to care
  - Patients less than 18 years old
  - Black patients
  - Elderly patients
6. According to the 2013 ACEP Clinical Policy regarding asymptomatic elevated blood pressure, which screening test would most likely affect disposition?
  - ECG
  - Creatinine
  - Troponin
  - None of these alters disposition
7. Which of the following management steps does the 2013 ACEP Clinical Policy recommend for asymptomatic markedly elevated blood pressure?
  - Screening for kidney disease
  - Referring for outpatient follow-up
  - Observation and repeat blood pressure measurement
  - Rapid lowering of mean arterial pressure at 25%

### Hypertensive Emergency TBL: Group Application Exercise (GAE)

**Digital:** https://forms.office.com/Pages/ShareFormPage.aspx?id=dGqfIFMJpE2HKkcxkbtXXbR94NwoakBJkvy9eJcaW6xUMU1ZR0xJNUszVkpGOTBCRFJMUjlIN1ZZUy4u&sharetoken=7wpTMKE2yTZSHzjERAoR

#### Case 1

A 55-year-old male with a past medical history of hypertension and ischemic cardiomyopathy with reduced ejection fraction presents with severe shortness of breath. His symptoms started abruptly 30 minutes ago. He is not having chest pain, but does complain of a cough. On exam, the patient appears to be in severe respiratory distress and is diaphoretic. He has rales in bilateral lung fields and 1+ edema in bilateral lower extremities.

Vitals: oral temperature 98.6 degrees Fahrenheit (°F), heart rate (HR) 130, blood pressure (BP) 200/150, pulse oximetry (SpO_2_) 86% on room air.

**Q1: Is this patient sick, not sick, or potentially sick?** [Fig f1-jetem-9-2-t1]

**Q2: What is your initial management, and what labs would you like to order?** [Fig f2-jetem-9-2-t1]

***Results:*** [Table t2-jetem-9-2-t1]

Troponin I < 0.1 ng/mL

B-type naturetic peptide (BNP) 800 pg/mL

Electrocardiogram (ECG): sinus tachycardia, nonspecific diffuse ST changes

**Q3: What imaging, if any, would you like to order?** [Fig f3-jetem-9-2-t1]

***Results:***

Bedside echocardiogram (ECHO): moderately decreased ejection fraction, positive for B-lines.

Chest X-Ray (CXR): cardiomegaly with diffusely increased interstitial markings.

Computed tomography (CT) or computed tomography angiography (CTA) of the chest: increased interstitial markings, no pulmonary embolism, consolidation, or dissection.

**Q4: At this point, what is your differential diagnosis for this patient and what do you think is most likely?** [Fig f4-jetem-9-2-t1]

**Q5: What is the treatment for this patient?** [Fig f5-jetem-9-2-t1]

#### Case 2

A 60-year-old-male with a past medical history of hypertension presents with chest pain radiating through to the back and left-sided weakness. His symptoms started abruptly thirty minutes prior to arrival. The patient endorses tobacco use and occasional cocaine use. On exam, he appears in distress, has 4 out of 5 weakness of the left lower and upper extremities and is otherwise neurologically intact.

Vitals: oral temperature 98.6 °F, HR 120, BP 186/104, RR 20, and SpO_2_ 95% on room air.

**Q1: Is this patient sick, not sick, or potentially sick?** [Fig f6-jetem-9-2-t1]

**Q2: What is your initial management, and what labs would you like to order?** [Fig f7-jetem-9-2-t1]

***Results:*** [Table t3-jetem-9-2-t1]

Troponin I <0.1 ng/mL

ECG: sinus tachycardia, nonspecific diffuse ST changes

**Q3: At this point, what is your differential diagnosis for this patient and what do you think is most likely?** [Fig f8-jetem-9-2-t1]

**Q4: What imaging, if any, and additional workup would you like to order?** [Fig f9-jetem-9-2-t1]

***Results:***

Computed tomography angiogram of the chest^5^

**Q5: What are the next steps, what are the blood pressure goals, and what BP lowering agents would you choose?** [Fig f11-jetem-9-2-t1]

#### Case 3

An 83-year-old female with a past medical history of diabetes, hypertension, coronary artery disease, and atrial fibrillation on warfarin presents with hemiparesis and confusion that started after acute onset of a severe headache. On exam, the patient is obtunded, not following commands, withdrawing to painful stimuli on the right side, and her pupils are equal and reactive to light.

Vitals: oral temperature 98.6°F, HR 120, BP 218/111, RR 20, SpO_2_ 95% on room air.

**Q1: Is this patient sick, not sick, or potentially sick?** [Fig f12-jetem-9-2-t1]

**Q2: What is your initial management, and what labs would you like to order?** [Fig f13-jetem-9-2-t1]

***Results:***

Fingerstick glucose: 120 mEq/L[Table t4-jetem-9-2-t1]

International normalized ratio (INR): 4.5

Troponin I: <0.1 ng/mL

ECG: sinus tachycardia, deep T-wave inversions

**Q3: At this point, what is your differential diagnosis for this patient and what do you think is most likely?** [Fig f14-jetem-9-2-t1]

**Q4: What imaging, if any, would you like to order?** [Fig f15-jetem-9-2-t1]

***Results:***

Computed tomography angiogram of the brain^6^

**Q5: What is the treatment for this patient, what are the BP goals, and what agents would you use?** [Fig f17-jetem-9-2-t1]

#### Case 4

An 80-year-old-female with a past medical history of hypertension is referred to the emergency department from clinic for elevated blood pressure. She has a moderate intensity, diffuse headache that started gradually today. She denies chest pain, shortness of breath, and confusion. She has not missed any doses of her anti-hypertensives. On exam, she appears to be in no acute distress and is neurologically intact with a steady gait.

Vitals: oral temperature 98.6°F, HR 65, BP 195/104, RR 20, SpO_2_ 95% on room air.

**Q1: Is this patient sick, not sick, or potentially sick?** [Fig f18-jetem-9-2-t1]

**Q2: What labs/imaging/meds/interventions would you like to order?** [Fig f19-jetem-9-2-t1]

**Q3: At this point, what is your differential diagnosis for this patient and what do you think is most likely?** [Fig f20-jetem-9-2-t1]

**Q4: Assuming your workup is negative, what is the treatment for this patient?** [Fig f21-jetem-9-2-t1]

## INSTRUCTOR MATERIALS

Answer keys to all exercises with explanations, are on the following pages.

Learners: please do not proceed.

### Hypertensive Emergency TBL: Readiness Assessment Test Key

1. The 2013 ACEP Clinical Policy regarding asymptomatic elevated blood pressure defines markedly elevated blood pressure as:
  - **Systolic blood pressure greater than or equal to 160 mmHg or diastolic blood pressure greater than or equal to 100 mmHg**
  - Systolic blood pressure greater than or equal to 180 mmHg or diastolic blood pressure greater than or equal to 110 mmHg
  - Systolic blood pressure greater than or equal to 220 mmHg or diastolic blood pressure greater than or equal to 180 mmHg
2. The 2013 ACEP Clinical Policy regarding asymptomatic elevated blood pressure states that routine screening for acute target organ injury (eg, serum creatinine, urinalysis, ECG) is not required.
  - **True**
  - False
3. According to the 2013 ACEP Clinical Policy regarding asymptomatic elevated blood pressure, routine ED medical intervention for markedly elevated blood pressure is:
  - Supported
  - **Not required**
  - Considered to have potential harm
4. According to the 2013 ACEP Clinical Policy regarding asymptomatic elevated blood pressure, rapid lowering of elevated blood pressure in the asymptomatic patient is:
  - Supported
  - Not required
  - **Considered to have potential harm**
5. The 2013 ACEP Clinical Policy recommends consideration of treating asymptomatic hypertension in all of the following populations except:
  - Patients with poor follow up
  - Patients with limited access to care
  - **Patients less than 18 years old**
  - Black patients
  - Elderly patients
6. According to the 2013 ACEP Clinical Policy regarding asymptomatic elevated blood pressure, which screening test would most likely affect disposition?
  - ECG
  - **Creatinine**
  - Troponin
  - None of these alters disposition
7. Which of the following management steps does the 2013 ACEP Clinical Policy recommend for asymptomatic markedly elevated blood pressure?
  - Screening for kidney disease
  - **Referring for outpatient follow-up**
  - Observation and repeat blood pressure measurement
  - Rapid lowering of mean arterial pressure at 25%

### Hypertensive Emergency TBL: Group Application Exercise Key (GAE Key)

#### Case 1

A 55-year-old male with a past medical history of hypertension and ischemic cardiomyopathy with reduced ejection fraction presents with severe shortness of breath. His symptoms started abruptly 30 minutes ago. He is not having chest pain, but does complain of a cough. On exam, the patient appears to be in severe respiratory distress and is diaphoretic. He has rales in bilateral lung fields and 1+ edema in bilateral lower extremities.

Vitals: oral temperature 98.6 degrees Fahrenheit (°F), heart rate (HR) 130, blood pressure (BP) 200/150, pulse oximetry (SpO_2_) 86% on room air.

**Q1: Is this patient sick, not sick, or potentially sick?**

- This patient is sick. He is tachycardic, tachypneic, hypertensive, and hypoxic (yikes!)

**Q2: What is your initial management, and what labs would you like to order?**

- Airway, breathing, circulation.
- Airway – Assess to ensure the patient’s airway is patent. The patient appears to be alert and oriented, but in distress.
- Breathing – Provide 100% oxygen (O2) via non-rebreather mask initially. Call respiratory therapy to set-up for bilevel positive airway pressure (BiPAP). This patient has a history of heart failure; thus, heart failure exacerbation should be on your differential.
- Circulation – At this time, the patient is hypertensive. 2 large-bore intravenous lines (IV) should be established immediately and should be placed on a cardiac monitor.
- Initial labs should include complete blood count (CBC), basic metabolic panel (BMP), troponin, B-type naturetic peptide (BNP), and an electrocardiogram (ECG).

***Results:*** [Table t5-jetem-9-2-t1]

1

Troponin I < 0.1 ng/mL

B-type naturetic peptide (BNP) 800 pg/mL

Electrocardiogram (ECG): sinus tachycardia, nonspecific diffuse ST changes

**Q3: What imaging, if any, would you like to order?**

Imaging should be considered in this patient. At minimum, a chest x-ray (CXR) should be obtained to assess for possible cardiomegaly, vascular congestion, pneumonia, or pneumothorax. A bedside cardiac and pulmonary ultrasound can also be considered to quickly assess left ventricular ejection fraction, right heart strain, and presence of B-lines (which may indicate fluid overload). Computed tomography (CT) or computed tomography angiography (CTA) of the chest may also be helpful but is not necessarily indicated unless aortic dissection or pulmonary embolism are high on the differential.

***Results:***

Bedside echocardiogram (ECHO): moderately decreased ejection fraction, positive for B-lines. Chest X-Ray (CXR): cardiomegaly with diffusely increased interstitial markings. Computed tomography (CT) or computed tomography angiography (CTA) of the chest: increased interstitial markings, no pulmonary embolism, consolidation, or dissection.

**Q4: At this point, what is your differential diagnosis for this patient and what do you think is most likely?**

- Given the patient’s history of ischemic cardiomyopathy and presentation of acute onset dyspnea, this patient is likely experiencing **acute decompensated heart failure with flash pulmonary edema.**
- There are several different clinical manifestations of acute decompensated heart failure (ADHF).
  1. ADHF with no cardiogenic shock or hypertensive crisis
  2. **ADHF with severe pulmonary edema** (in this patient’s case)
  3. ADHF with cardiogenic shock
  4. ADHF secondary to high-output heart failure
  5. ADHF secondary to right-sided heart failure
  ○ ADHF with severe pulmonary edema is characterized by the rapid onset of symptoms, typically with a systolic blood pressure > 180 mmHg.^7^ This condition is typically caused by poorly controlled hypertension. Elevated blood pressure can cause acute increases in afterload, leading to pulmonary edema and poor systemic perfusion. Decreased perfusion subsequently results in a sympathetic surge that further increases afterload and causes rapid decompensation.
- Major causes of ADHF should also be considered, such as myocardial infarction, arrythmias, acute valve syndromes, septal rupture, and cardiomyopathic states.
- Other causes of pulmonary edema include noncardiogenic causes, such as hypertensive emergency, upper airway obstruction, neurogenic cases, iatrogenic fluid overload, and ARDS.

**Q5: What is the treatment for this patient?**

Recommended by the Heart Failure Society of America, the initial management of ADHF with severe pulmonary edema is focused on hemodynamic stabilization, support of oxygenation and ventilation, and providing symptom relief.^8^ This is achieved by focusing interventions on vasodilating arterial circulation while maintaining oxygenation.

In general, for treatment of acute heart failure, consider the mnemonic “PPV HAVoC.” 9

- **PPV** = “positive pressure ventilation.” Non-invasive PPV reduces cardiac preload (by increasing intrathoracic pressure) and reduces afterload
  ○ BiPAP
  ○ Begin with positive end-expiratory pressure (PEEP) of ~8cm H20 and slowly titrate upward
- **H**ypotension correction (not indicated in this patient)
- **A**fterload reduction in patients who are HYPERtensive. Nitroglycerin is the first-line medication in ADHF without cardiogenic shock. It reduces afterload and preload.
  ○ High-dose nitroglycerin. Start with 3 sprays sublingually (400mcg per dose)
  ○ Start a nitroglycerin infusion at 50–100mcg/min and titrate up by 20mcg/min every 10 minutes to 100–200mcg/min.^10^
  ○ Continue titration until resolution of symptoms or SBP < 160 mmHg
  ○ At higher doses, nitroglycerin has greater vasodilation > venodilation
- **V**olume status (diuresis vs. intravascular volume repletion)
  ○ IV furosemide 1–2x the patient’s daily home dose should be administered
  ○ Diuresis typically occurs 30–60 minutes after initial IV administration
- **C**ause (treat underlying cause)

This patient’s disposition should ultimately be an admission to telemetry level of care at minimum.

#### Case 2

A 60-year-old-male with a past medical history of hypertension presents with chest pain radiating through to the back and left-sided weakness. His symptoms started abruptly thirty minutes prior to arrival. The patient endorses tobacco use and occasional cocaine use. On exam, he appears in distress, has 4 out of 5 weakness of the left lower and upper extremities and is otherwise neurologically intact.

Vitals: oral temperature 98.6 °F, HR 120, BP 186/104, RR 20, and SpO_2_ 95% on room air.

**Q1: Is this patient sick, not sick, or potentially sick?**

This patient is sick. His vitals are remarkable for tachycardia and hypertension with obvious distress and focal weakness on exam.

**Q2: What is your initial management, and what labs would you like to order?**

- Airway, breathing, circulation.
- Airway – Assess to ensure the patient’s airway is patent. The patient appears to be alert and oriented, but in distress.
- Breathing – The patient is saturating well on room air
- Circulation – The patient is hypertensive. 2 large-bore IV’s should be established immediately and should be placed on a cardiac monitor.
- Initial labs should, at minimum, include CBC, BMP, troponin, and an ECG. D-dimer can also be considered.

***Results:*** [Table t6-jetem-9-2-t1]

Troponin I <0.1 ng/mL

ECG: sinus tachycardia, nonspecific diffuse ST changes

**Q3: At this point, what is your differential diagnosis for this patient and what do you think is most likely?**

- Emergent causes of chest pain
  ○ Acute coronary syndrome (ACS)
  ○ Aortic dissection
  ○ Coronary artery dissection
  ○ Myocardial rupture
  ○ Esophageal perforation
  ○ Pulmonary embolism
  ○ Tension pneumothorax
- The differential diagnosis for chest pain is broad. However, given the patient’s history of hypertension, tobacco and cocaine abuse, and his presentation with acute neurologic deficit, aortic dissection should be high on the differential.
- The Aortic Dissection Detection Risk Score (ADD-RS) should be used to guide workup of low to moderate risk patients for whom acute aortic syndromes are in the differential diagnosis. One point should be added for each of the three categories that contain at least one feature.^11^
  ○ Any high risk condition
    ▪ Marfan syndrome, family history of aortic disease, known aortic valve disease, recent aortic manipulation, or known thoracic aortic aneurysm
  ○ Any high risk pain feature
    ▪ Chest, back, or abdominal pain described as abrupt onset, severe intensity, or ripping/tearing
  ○ Any high risk exam feature
    ▪ Evidence of perfusion deficit (pulse deficit, systolic blood pressure differential, or focal neuro deficit plus pain), new aortic insufficiency murmur (with pain), hypotension/shock
  ○ Scoring
    ▪ 0 = low risk
    ▪ 1 = intermediate risk
    ▪ > 1 = high risk
- Workup based on risk category
  ○ Low risk and intermediate risk
    ▪ Obtain CXR, look for mediastinal widening, left sided pleural effusion, or widening of aortic contour.
    ▪ Proceed to D-dimer testing. If < 500 ng/mL, consider stopping dissection workup, or if >500 ng/mL, consider CTA.
  ○ High risk
    ▪ Consider proceeding directly to CTA or other conclusive imaging

**Q4: What imaging, if any, and additional workup would you like to order?**

- Patient should get a CXR and CTA aortogram of the chest.
- Bedside ultrasound could also assist in quickly ruling in patients when aortic outflow tract is > 4cm
  ○ Assess for pericardial effusion and tamponade as a dissection can dissect all the way to the pericardium.
- ECG’s findings can include left ventricular hypertrophy, inferior ischemia, or non-specific ST-T changes.

***Results:***

Computed tomography angiogram of the chest^5^

**Q5: What are next steps, what are the blood pressure goals, and what BP lowering agents would you choose?**

The patient has an aortic dissection. Arterial dissection occurs between the intima and adventitia of arteries, allowing blood to enter the media. Aortic dissections are classified into Stanford Type A and Type B.

- Type A (proximal) involves any portion of the ascending aorta
- Type B (distal) is isolated to the descending aorta

The mainstay of treatment is centered around decreasing shearing forces on the aorta.^11^

- Heart rate goal < 60 bpm
- Blood pressure SBP < 100–110 mmHg
- **Start negative chronotropic agent prior to controlling blood pressure to avoid reflex tachycardia**

**Heart Rate Control (beta blockers or calcium channel blockers)**

- Esmolol drip (short half-life and easy to titrate)^12^
  ○ Bolus 0.5mg/kg over one minute
  ○ Infuse 0.05mg/kg/min (titrate upward in 0.05mg/kg/min increments to a maximum of 0.3mg/kg/min)
- Labetalol, metoprolol, and diltiazem are also acceptable options

**Blood Pressure Control**

- Nicardipine drip (short half life, easy to titrate)^12^
  ○ Start infusion at 5mg/hr
  ○ Titrate upward by 2.5mg/hr every 10min until goal blood pressure
- Clevidipine or nitroprusside infusions are also acceptable options

Analgesia is also helpful in blood pressure and catecholamine reduction. Consider fentanyl/morphine.

**Emergent Surgical Consultation**

- Type A dissections require surgery. Consult cardiothoracic surgery. Consider vascular surgery if extension into descending aorta.
- Type B dissections are primarily medical management with vascular surgery consultation.

#### Case 3

An 83-year-old female with a past medical history of diabetes, hypertension, coronary artery disease, and atrial fibrillation on warfarin presents with hemiparesis and confusion that started after acute onset of a severe headache. On exam, the patient is obtunded, not following commands, withdrawing to painful stimuli on the right side, and her pupils are equal and reactive to light.

Vitals: oral temperature 98.6°F, HR 120, BP 218/111, RR 20, SpO_2_ 95% on room air.

**Q1: Is this patient sick, not sick, or potentially sick?**

This patient is sick. She is tachycardic and hypertensive. Her Glasgow Coma Scale (GCS) is 6 (eyes opening = 1, verbal response = 1, motor response = 4).

**Q2: What is your initial management, and what labs would you like to order?**

- Airway, breathing, circulation.
- Airway – The patient’s GCS is 6. Given her severe altered mental status, she is unable to protect her airway and should be intubated.
- Breathing – The patient is saturating well on room air, but is not protecting her airway.
- Circulation – The patient is hypertensive. 2 large-bore IV’s should be established immediately and should be placed on a cardiac monitor.
- Given her hemiparesis and anticoagulated status, hemorrhagic stroke should be high on the differential. Initial labs should include a point-of-care glucose, CBC, metabolic panel, troponin, coagulation times, drug screen, and ECG.

***Results:***

Fingerstick glucose: 120 mEq/L[Table t7-jetem-9-2-t1]

International normalized ratio (INR): 4.5

Troponin I: <0.1 ng/mL

ECG: sinus tachycardia, deep T-wave inversions

**Q3: At this point, what is your differential diagnosis for this patient and what do you think is most likely?**

- Stroke-like symptoms
  ○ Hemorrhagic stroke
  ○ Ischemic stroke
  ○ Seizures/postictal paralysis
  ○ Hypoglycemia
  ○ Hyponatremia
  ○ Meningitis
  ○ Drug toxicity
  ○ Cerebral sinus thrombosis
  ○ Bell’s Palsy
  ○ Conversion disorder
- The differential for stroke-like symptoms is broad. However, this patient has multiple high-risk comorbidities for a stroke and is presenting with depressed GCS and hemiparesis. Hemorrhagic stroke should be high on the differential, especially given her anticoagulated status because warfarin use is a significant risk factor.
- Hemorrhagic strokes are often clinically indistinguishable from ischemic strokes.
  ○ ECG’s may show deep T-wave inversions and prolonged QT intervals.

**Q4: What imaging, if any, would you like to order?**

- CT head (non-contrast) should be ordered. In acute intracerebral hemorrhage, sensitivity is 95–100%.^13^
- Can also consider CTA brain/neck to assess for vascular abnormalities
- CXR may be helpful in ruling out infection

***Results:***

Computed tomography angiogram of the brain^6^

**Q5: What is the treatment for this patient, what are the BP goals, and what agents would you use?**

This patient has an intracerebral hemorrhage (ICH). Mainstay of treatment is focused on decreasing intracerebral pressure and reversing coagulopathy. Don’t forget your ABC’s because this patient should be intubated for airway and neuroprotection.

- Elevate head of bed to 30 degrees
- Seizure prophylaxis is NOT recommended according to guidelines from the American Heart Association.^14^

**Blood Pressure Control**

- Rapid SBP lowering < 140 mmHg within one hour of presentation has been advocated in early research, but recent studies have shown no clinical difference in outcome between SBP < 140 and <180 mmHg.^15^–^17^
- If presenting SBP between 150–220 mmHg, lower to target of 140 mmHg.
- If presenting SBP > 220 mm Hg, lower to target range of 140 – 160 mmHg.^14^
- Nicardipine drip (fast-acting and easily titratable)^12^
  ○ Start infusion at 5mg/hr
  ○ Titrate upward by 2.5mg/hr every 10min until goal blood pressure
- Consider labetalol pushes for SBP < 160 mmHg for ease of administration and long duration of effect^18^
  ○ 20mg bolus over 1–2 minutes, repeat every 3–5 minutes until target blood pressure

**Reverse Coagulopathy**

- Warfarin reversal^19^, ^20^
  ○ Vitamin K – 2.5–10mg IV depending on INR. Administer single dose over 10–20 minutes.
  ○ 4-factor prothrombin complex concentrate (preferred over fresh frozen plasma because PCC contains higher amounts of clotting factors and can be administered much more quickly)
- Direct-thrombin-inhibitor reversal^21^
  ○ Idarucizumab – 5g IV push
  ○ 4-factor PCC if unavailable
- Factor-Xa-inhibitor reversal^21^
  ○ Andexanet alpha
  ○ 4-factor PCC if unavailable
- Aspirin, clopidogrel^21^
  ○ Desmopressin – 0.3mcg/kg
  ○ Consider platelet transfusion ONLY if thrombocytopenic < 50,000

**Admit for acute or subacute ICH.**

Consult neurosurgery for consideration of hematoma evacuation or external ventricular drain.

#### Case 4

An 80-year-old-female with a past medical history of hypertension is referred to the emergency department from clinic for elevated blood pressure. She has a moderate intensity, diffuse headache that started gradually today. She denies chest pain, shortness of breath, and confusion. She has not missed any doses of her anti-hypertensives. On exam, she appears to be in no acute distress and is neurologically intact with a steady gait.

Vitals: oral temperature 98.6°F, HR 65, BP 195/104, RR 20, SpO_2_ 95% on room air.

**Q1: Is this patient sick, not sick, or potentially sick?**

This patient is not sick. Some may argue that given her elevated blood and associated headache, the patient is potentially sick. However, according to the American College of Emergency Physicians, for patients aged 18 years or older who present to the ED with elevated blood pressure and *without* signs and symptoms of acute organ injury, routine screening and treatment of asymptomatic hypertension is not required, if they have good follow-up with a primary care provider. However, in patient populations with poor follow-up, screening and acute interventions may be indicated.

According to the Joint National Committee on Prevention, Detection, Evaluation and Treatment of High Blood Pressure (JNC 7), stage 2 hypertension is defined as systolic blood pressure greater than or equal to 160 mmHg or diastolic blood pressure greater than or equal to 100 mmHg.^22^

“Asymptomatic,” in this case, refers to the absence of signs of acute organ injury. “Asymptomatic” patients may still have headache, lightheadedness, nausea, palpitations, epistaxis, or anxiety; however, these are not signs of end organ damage.^23^

**Q2: What labs/imaging/meds/interventions would you like to order?**

No labs or interventions are needed. This patient’s history and physical exam are reassuring. In patients presenting with elevated BP, history and physical exam should focus on searching for signs of end-organ damage, paying particular attention to neurologic, cardiovascular, and pulmonary exams.

Alarm signs of hypertensive emergency include, but are not limited to:

○ Chest pain
○ Respiratory distress, hypoxia
○ Focal neurologic deficits
○ Loss of vision
○ Altered mental status, seizures
○ Severe headache (think: “thunderclap”)
○ Oliguria

In patients who complain of flank pain or oliguria, or in populations with poor follow-up, screening for an elevated creatinine level to identify kidney injury may be indicated.

**Q3: At this point, what is your differential diagnosis for this patient and what do you think is most likely?**

Before diagnosing a patient with asymptomatic hypertension, end-organ dysfunction or emergent causes of a patient’s hypertension must be ruled-out.

Differential diagnoses:

○ ACS
○ Aortic dissection
○ Flash pulmonary edema
○ Hypertensive encephalopathy
○ Hemorrhagic/ischemic stroke
○ Acute kidney injury

**Q4: Assuming your workup is negative, what is the treatment for this patient?**

Patients with asymptomatic markedly elevated blood pressure should be referred for outpatient follow-up. In populations who have poor follow-up, emergency physicians may treat markedly elevated blood pressures in the ED and/or start that patient on long-term therapy.^22^

It is important to note that providers may sometimes feel pressured to correct an asymptomatic patient’s elevated blood pressure by administering intravenous antihypertensive medications (IVAH). However, the use of IVAH based purely on numerical value and without any supporting evidence of their use may be harmful and has no proven value.^24^, ^25^
